# Thoracoscopic splanchnicectomy as a palliative procedure for pain relief in carcinoma pancreas

**DOI:** 10.4103/0972-9941.55106

**Published:** 2009

**Authors:** Arun Prasad, Piush Choudhry, Sunil Kaul, Gaurav Srivastava, Mudasir Ali

**Affiliations:** Indraprastha Apollo Hospital, Department of General and Minimal Access Surgery, Department of Surgical Sciences, New Delhi, India

**Keywords:** Cancer, pain, palliation, pancreas, thoracoscopy, splanchnicectomy, VATS

## Abstract

Thoracoscopic splanchnicectomy has been used for the management of upper abdominal pain syndromes as an alternative to celiac plexus block for conditions such as chronic pancreatitis or supramesocolic malignant neoplasms, including unresectable pancreatic cancer. This procedure is similar to the percutaneous block with a higher degree of precision and avoids the side effects associated with the local diffusion of neurolytic solutions. Thoracoscopic splanchnicectomy appears to be a better treatment in such cases as the procedure is done under direct vision and less dependent on anatomical variations.

## INTRODUCTION

With limitations of the celiac plexus block and less associated morbidity with this procedure, thoracoscopic splanchnicectomy appears to be the surgery for palliation for conditions such as chronic pancreatitis or supramesocolic malignant neoplasms, including unresectable pancreatic cancer.[1] The first reported laparoscopic splanchnicectomy was performed in 1943 by Mallet-Guy, while excision of supraphrenic part of celiac nerve from thoracotomy access was reported by Ston and Chauvin (1990) and later by Melki *et al.* (1993) pioneered in thoracoscopic splanchnicectomy. Few cases have been reported though with encouraging results. Celiac plexus is the largest sympathetic plexus located at L1 level posterior to the vena cava on the right side and just lateral to the aorta on the left side. The plexus is composed of a dense network of interconnecting presynaptic sympathetic nerve fibres derived mainly from the greater (T5T9), lesser (T10–T11) and least (T12) splanchnic nerves. It also receives parasympathetic fibres from the vagus nerve. Splanchnic nerve neurolysis or neurolytic celiac plexus block for the treatment of chronic abdominal pain was first reported by Kappis in 1914. In 1918, Wendling was the first to report an anterior transabdominal technique, and the techniques were improvised in tune with the radiological advances but the complications associated with the procedure often outweighs the benefits. Thoracoscopic splanchnicectomy is yet in the incipient stages of minimal access procedures and further data are required before it can be documented as a procedure for palliative surgery.

## CASE REPORT

A 64-year-old male presented with complaints of pain upper abdomen on and off since last 4 months. Pain was moderate to severe in intensity, radiating to back and relieved by bending forward. There was loss of weight and appetite. No other significant history of medication or hospitalization was found.

CT scan of the abdomen done on 28.08.08 revealed pancreatic body mass lesion with extension into peripancreatic fat encasing adjacent vessels. CT guided FNAC was suggestive of adenocarcinoma pancreas.

During surgery the tumour was found to be arising from the body and tail of pancreas infiltrating into the duodenojejunal flexure, roof of mesocolon and encasing the middle colic and SMV, thus making the tumour unresectable.

**Figure 1:**
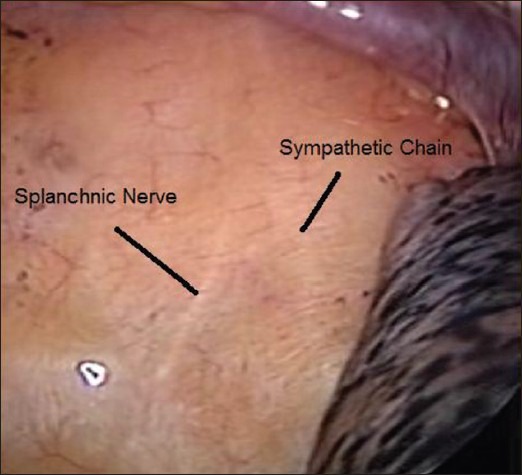
Thoracoscopic view of nerves

**Figure 2:**
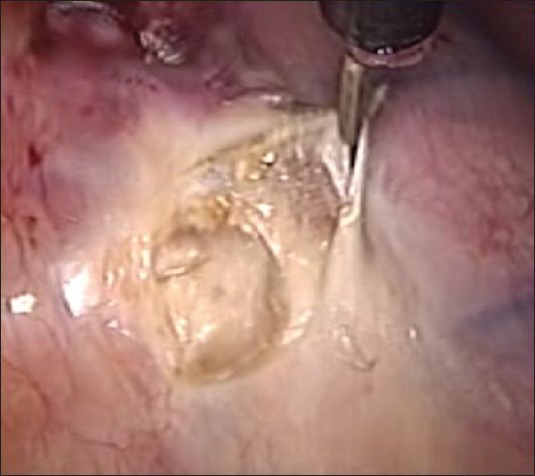
Nerve being cut with cautery

The patient was referred to the pain management clinic where splanchnicectomy was advised. Bilateral thoracoscopic splanchnicectomy was performed with 10 mm port in the sixth ICS space for the telescope and two 5 mm ports made in the second ICS and fourth ICS anteriorly. The sympathetic trunk was identified and the greater splanchnic nerve seen at the T8 level crossing over towards the right crus of the oesophageal opening into the diaphragm [Figure 1]. The splanchnic nerve was divided using cautery together with its connections to the main sympathetic trunk [Figure 2]. Similar procedure was repeated on the left side.

The patient reported less pain on visual analogue scale VAS 2/10 as compared to preoperative VAS 8/10 in the immediate postoperative period; the patient is now relieved and is on mild analgesics.

## DISCUSSION

Celiac plexus blocks have been used for the treatment of chronic abdominal pain for nearly a century now, and the excision of nerve fibres has been shown to decrease the pain significantly. Even though these techniques have been improvised with the advances in the field of radiology, there have been documented evidences of complications including sexual dysfunction, pneumothorax, retroperitoneal haemorrhage, retroperitoneal abscess, kidney damage, rarely paraplegia besides postural hypotension and diarrhoea.[2] Besides these complications, distorted anatomical relationships due to ascites, organomegaly, tumour bulk, obesity, peritoneal seeding and previous surgery limit the procedure. Moreover, these procedures had to be repeated a number of times to achieve optimal results which increased the risk of iatrogenic complications.

Thoracoscopic splanchnicectomy is a percutaneous procedure performed under direct vision. The advantages include that the procedure can be performed bilateral in the same prone position, higher precision video assisted identification and division of all the roots of the splanchnic nerves, from T5 through T12 can be performed, no need for selective double-lung ventilation to create the working space and use of CO_2_ insufflation usually allows to perform a two trocar technique. It also avoids the side effects associated with the local diffusion of neurolytic solutions. The limitations include difficulty in pleural adhesions and the likelihood that pain relief reduces with increased period of survival in cancer patients.

Whether unilateral procedure is sufficient[3] or neurotomies would depend on the side of pain radiation, whether denervation of the nerve roots or just the splanchnic nerve is sufficient[4] are questions to be answered in the future, but certainly the procedure is a valuable attempt to provide palliation in patients. This procedure has been reported with either few or no complications. Kordiak *et al.* (2005)[5] in a study of 31 patients reported no complications of thoracoscopic splanchnicectomy surgery in any of the study patients within the observation time period and stated its use as current palliative analgesic therapy. The patients referred for palliative surgery have on an average 6 months survival time, and subjecting these for procedures that can become an important source of morbidity is not justified.
